# VISTA Alleviates Microglia-Mediated Neuroinflammation After Cerebral Ischemia–Reperfusion Injury via Regulating ACOD1/Itaconic Acid Metabolism

**DOI:** 10.1007/s12035-025-05106-x

**Published:** 2025-06-19

**Authors:** Yilei Sun, Dan Liu, Yanchen Liu, Lijun Chi

**Affiliations:** Department of Neurology, The First Affiliated Hospital of Harbin Medical University, Harbin, China

**Keywords:** Cerebral ischemia–reperfusion injury, Immune checkpoint, VISTA, ACOD1, TCA cycle

## Abstract

Cerebral ischemia–reperfusion injury (CIRI) induces significant microglial inflammation. V-type immunoglobulin domain–containing suppressor of T cell activation (VISTA), a novel inhibitory immune checkpoint, participates in myeloid cell metabolism. This study aims to investigate the molecular mechanisms of VISTA’s protective effects on CIRI by modulating microglial metabolism. In this study, differentially expressed genes (DEGs) were extracted from GSE77986 to identify hub gene VISTA. Transient middle cerebral artery occlusion (tMCAO) and oxygen–glucose deprivation and reoxygenation (OGD/R) were conducted to mimic CIRI. AAVMG1.2-VSIR was injected intracerebroventricularly into Cx3cr1^Cre^ mice, while over-expression plasmids were transfected into BV2 to intervene VISTA. The mice underwent LONGA scoring, H&E, Nissl, and TTC staining. Western blot and qRT-PCR were conducted for VISTA, IL-6, TNFα, IL-1β, and IL-10. Microglial proliferation was assessed by Edu staining and CCK8. RNA-sequencing (RNA-seq) analysis was used to investigate downstream pathways. Tricarboxylic acid (TCA) cycle intermediates were measured using ELISA. ACOD1/IκBα/NF-κB pathway was validated by Western blot. Eight DE-ICGs were identified through differential analysis, with VSIR exhibiting the highest expression. Additionally, VISTA was found decreased in microglia around the infarction site. Compared with CIRI group, VISTA reduced the infarct volume, improved neurological deficit, and decreased IL-6, TNFα, and IL-1β, while increasing IL-10, and suppressing microglia proliferation. RNA-seq showed that the DEGs primarily participated in microglial glucose metabolism and the IκBa/NF-κB pathway. VISTA promoted ACOD1 expression and itaconate (ITA). The protective function on CIRI and inhibitory effect on IκBa/NF-κB of VISTA were abrogated by ACOD1 knockdown.

**Supplementary Information:**

The online version contains supplementary material available at 10.1007/s12035-025-05106-x.

## Introduction

Acute ischemic stroke (AIS) is a leading cause of death and disability globally [1]. It results from a sudden reduction in brain blood flow, leading to cerebral tissue and cell damage [2]. The primary therapeutic objective for AIS patients is the prompt and effective recanalization of occluded blood vessels through thrombolytic therapy or endovascular thrombectomy [3]. Rapid reperfusion is known to induce cerebral ischemia–reperfusion injury CIRI [4]. However, the pathogenesis of CIRI is complex and not fully understood. The inflammatory cascade reaction plays a crucial role in this process [5]. Investigating the immune-inflammatory response may shed light on the mechanisms of neuronal damage and inform effective interventions. Microglia, as brain-resident macrophage, constantly monitor the cerebral environment to maintain normal brain function and homeostasis [6]. Upon CIRI, resting microglia become activated, migrating to ischemic and penumbral regions to either exert neuroprotective effects or initiate inflammatory responses. Thus, targeting neuroinflammatory responses of activated microglia presents a potential therapeutic strategy [7].

Tricarboxylic acid (TCA) cycle is a primary glucose metabolic pathway that fuels cellular processes [8]. Immune cells undergo glucose metabolic reprogramming upon activation [9, 10]. Understanding different metabolic pathways in immune cells is crucial for influencing inflammatory responses. Aconitate decarboxylase 1 (ACOD1, also known as IRG1) produces itaconate, a TCA cycle metabolite recently recognized for its role in modulating the TCA cycle and inflammatory responses in ischemia [11]. ACOD1 exhibits anti-inflammatory effects through multiple mechanisms, with ACOD1 knockout (KO) showing a significant increase in lesion volume in transient middle cerebral artery occlusion (tMCAO) mice [12, 13]. However, the underlying mechanisms remain largely elusive. Targeting microglial glucose metabolic intermediates could provide a novel approach to inflammation regulation.

Immune checkpoints comprise receptors and ligands expressed by antigen-presenting cells (APCs) and T cells. They regulate T cell activation by providing costimulatory and coinhibitory signals, crucial for immune balance [14]. The V-type immunoglobulin domain–containing suppressor of T cell activation (VISTA), encoded by VSIR, is a negative checkpoint regulator (NCR) predominantly found on myeloid and T cells [15, 16]. VISTA plays a role in maintaining T cell quiescence and inhibiting T cell activation. Previous studies have shown that VISTA induces tolerance and anti-inflammatory programs in macrophages and modulates the extent of innate inflammation in vivo [17]. In the central nervous system (CNS), VISTA, expressed by microglia, decreases during neuroinflammation [18]. Differential expression of VISTA has been observed in various stages of multiple sclerosis (MS) lesions. VISTA negatively regulates microglia glycolysis and activation in sepsis-associated encephalopathy [19]. VISTA KO affects genes related to microglial proliferation, immune activation, and phagocytic capability in vitro [20]. More importantly, VISTA mediates macrophage reprogramming via ACOD1 [17]. Yet, the role of VISTA in CIRI has not been extensively studied.

In the present study, a tMCAO model and an oxygen–glucose deprivation and reoxygenation (OGD/R) model were well established to mimic CIRI. The AAVMG1.2-VSIR with Cre-Loxp system in vivo and plasmid transfection in vitro were constructed to interfere VISTA respectively. The purpose of the study was to explore the molecular mechanisms underlying the protective effects of VISTA following CIRI by regulating microglia metabolic intermediates. The study on immunometabolism response may help elucidate further explore effective interventions.

## Materials and Methods

### Animals

Male C57BL/6 mice weighing 22–25 g (aged 8–10 weeks) were purchased from Changsheng (Liaoning, China). Cx3cr1^Cre^ mice in C57BL/6 strain background were obtained from Cyagen (Jiangsu, China). All male Cx3cr1^Cre^ mice involved in the experiments were heterozygous. The mouse breeding method involved positive cross-mating. To confirm the presence of the Cre sequence, genomic DNA extracted from the tail was amplified using the following primers [21] (Table 1, Fig. S1).

**Table 1:** The primer sequences for genomic DNA extracted from the tail of Cx3cr1^Cre^ mice

Primers for Region1 PCR:	Mutant: 508 bp
P1:	5′-GGATGAGTGAAGACAAAATCTAGTTCC-3′
P2:	5′-CTTGCAGGTACAGGAGGTAGTCC-3′
Primers for Region2 PCR:	Wild type: 307 bp
P1:	5′-GGATGAGTGAAGACAAAATCTAGTTCC-3′
P3:	5′-CCTCAGCAGAATCGTCATACTCAA-3′

All experimental mice were managed in strict accordance with the experimental animal management guidelines of the First Affiliated Hospital of Harbin Medical University and the US National Institutes of Health’s recommendations. The mice were housed in a controlled environment (20 ± 2 °C) under a 12-h light–dark cycle with ad libitum access to food and water.

### TMCAO Surgery

The CIRI model was induced by an 1 h tMCAO, following established methods [22]. Mice were anesthetized with 3% isoflurane, and their right common, internal, and external carotid arteries were isolated. The right external carotid artery was ligated, and a 0.21-mm silicon-coated filament (602156PK5Re, Doccol Co., USA) was inserted into the internal carotid artery through the external carotid artery to occlude the middle cerebral artery, reaching approximately 10 mm at the bifurcation. After 1 h of ischemia, the filament was withdrawn to allow reperfusion. The following two experiments were conducted independently: (1) VISTA in the peri-infarct cerebral tissue collected at 1, 3, and 7 days post-tMCAO. (2) We studied the effects of VISTA on the activated microglia-mediated inflammatory brain damage after tMCAO. The mice were randomly divided into the following groups: Sham group; tMCAO group, undergoing tMCAO surgery; tMCAO + VISTA group, injected with AAVMG1.2-VSIR post tMCAO surgery; tMCAO + vector group, injected with AAVMG1.2-NC post tMCAO surgery. Each experiment was performed at least three times.

### Intracerebroventricular Injection of AAV

VSIR or NC sequence was inserted into an AAVMG1.2 construct containing Loxp and EGFP (GeneChem, Shanghai, China). For conditional overexpression experiments of VSIR in microglia, the virus (1E + 13v.g/ml) was injected intracerebroventricularly (I.C.V.) into Cx3cr1^Cre^ mice using a previously described method [23–25]. The injection site, relative to the bregma, was anteroposterior 1 mm, right lateral 1.5 mm, and depth 2.0 mm. A 5-µl solution was delivered into the ipsilateral ventricle and administered 10 min, followed by a slow 5-min withdrawal of the needle. The burr hole was sealed with bone wax post-injection. Samples were collected 5 days later for experiments [26]. VISTA was significantly increased in microglia post-injection, demonstrating the success of the AAV intervention (Fig.S2).

### Neurological Function Assessment

All mice were undergoing Longa’s neurological severity scale evaluation by a blinded inspector. Deficits were scored as follows: 0, no neurological deficits; 1, inability to fully extend the forelimbs and contralateral body; 2, circling to contralateral surgical side; 3, falling to the contralateral surgical side; 4, inability to walk spontaneously and depressed consciousness [27]. The mice with scores of 1–4 were classified as valid tMCAO model [28]. Each test was repeated three times, and the average score was used for evaluation.

### 5-Triphenyl Tetrazolium Chloride Staining

2,3,5-Triphenyl tetrazolium chloride (TTC) staining was employed to determine cerebral infarction area. Coronal brain sections were immersed in 2% TTC solution (G3005, Solarbio, China) at 37 °C for 30 min. The total and infarcted brain area were quantified using Image J software. Infarct volume was calculated as (infarcted area/total brain area) × 100%.

### Immunohistochemistry Assays

Brain tissues were fixed, paraffin-embedded, and sectioned into 4-µm slices. Sections were dewaxed, dehydrated, and incubated in 3% hydrogen peroxide for antigen retrieval. The slices were heated in 0.01 M sodium citrate buffer (pH 6.0) and then cooled in deionized water. Ten percent of normal goat serum (SL038, Solarbio, China) was used to block non-specific protein binding. The slices were incubated with the primary VISTA (1:300, 54,979, CST, USA) antibody overnight at 4 °C, followed by incubation with the secondary antibody (PV6001, ZSGB-BIO, China) for 60 min at room temperature. Antibody binding was visualized using a diaminobenzidine kit. The positive areas were visualized by light microscopy.

### Immunofluorescence

Brain tissues sections were mounted and permeabilized with 1% Triton X-100 for 10 min, and blocked for 1 h at 37 °C in 10% normal goat serum (NGS). Antibody incubations were performed in 5% NGS. The primary antibodies were rat anti-Iba1 (1:200, ab283346, Abcam), rabbit anti-VISTA (1:200, ab300042, Abcam), rabbit anti-CD16 (1:200, 16,559–1-A, Protintech) and rabbit anti-CD206 (1:200, 18,704–1-AP, Protintech). The secondary antibodies were Alexa Fluor 488 labeled Goat anti-rat IgG (1:200, RS23240, Immunoway) and Alexa Fluor 594 labeled Goat anti-rabbit IgG (1:200, SA00013-4, Protintech). Image acquisition was performed using Nikon A1 system (Nikon; Tokyo, Japan).

### Histological Analysis

Brain tissue was conducted the same as above. For H&E staining, the sections were stained with hematoxylin and eosin according to standard protocols [29]. For the purpose of detecting the neuronal loss via Nissl staining, the sections were subjected to staining for 30 min utilizing cresyl violet solution (0.25%) at ambient temperature, followed by stepwise dehydration with 95 and 100% ethanol in water, and clearing using xylene [30]. The images were subsequently evaluated utilizing NIS-Elements D (version 5.0). At least three views from the central and peripheral areas were selected for photography and analysis.

### Cell Culture Conditions and OGD/R Model

Mouse BV2 microglial cells (Procell, Hubei, China) were cultured in Dulbecco’s Modified Eagle’s Medium (DMEM, Gibco, New York, USA) supplemented with 10% fetal bovine serum (Gibco, New York, USA) and 1% penicillin–streptomycin (Hyclone, Logan, UT, USA). They were maintained at 37 °C in a humidified 5% CO_2_ humidified atmosphere, with medium changes every 2 days [31]. The BV2 cells underwent OGD/R to mimic cerebral ischemia in vitro as previously reported [32]. The cells were incubated in glucose-free DMEM (Procell) in an atmosphere of 5% CO_2_ and 95% N_2_ for 2 h, 4 h, and 6 h, followed by reoxygenation in complete medium for 24 h. Microglial amoeboid morphology was observed, and the upregulation of pro-inflammatory cytokines was validated, thereby confirming the successful establishment of the OGD/R model. The following three experiments were conducted: (1) to validate the levels of VISTA on BV2, cells were collected at OGD2, 4 and 6 h with reperfusion 24 h. (2) We further investigated the effect of VISTA-mediated microglial function post OGD/R. The groups include the control group; the OGD/R group, undergoing OGD 4 h/R24 h; OGD/R + VISTA, undergoing VSIR overexpression plasmids transfection before OGD 4 h/R24 h; and OGD/R + vector, undergoing PCDNA3.1 transfection before OGD 4 h/R24 h. (3) To confirm the effect of ACOD1 on VISTA, the groups include the control group; OGD/R + oe-VISTA, undergoing VSIR overexpression plasmids transfection before OGD/R; OGD/R + oe-VISTA + si-ACOD1, undergoing VSIR overexpression plasmids and si-ACOD1 transfection before OGD/R; and OGD/R + oe-VISTA + si-NC, undergoing VSIR overexpression plasmids and si-NC transfection before OGD/R.

### Plasmids and siRNA Transfection

VSIR overexpression plasmids, using the PCDNA3.1 as vector, and small interfering RNAs (siRNAs) targeting ACOD1 were acquired from GeneCreate (Wuhan, China). These agents were transfected into BV2 cells using Lipofectamine 2000 transfection reagent (11,668,500, Thermo Fisher Scientific) following a previously described method [33]. After 48 h, the cells were used for further experiments.

### Detection of Citric Acid, Isocitric Acid, Cisaconite Acid, and Itaconic Acid

Activity test kits for citric acid, isocitric acid, and cisaconitic acid, and an ELISA kit for ITA, were sourced from MLBIO (Shanghai, China). The procedures followed the manufacturer’s instructions. Post-treatment, 500 × 10^4^ BV2 cells were collected for citric acid, isocitric acid, and cisaconitic acid detection. The supernatants, obtained after extraction, ultrasonic disruption, and centrifugation, were analyzed using an Enzyme Label Analyzer. Citric acid was detected at 545 nm, while isocitric acid and cisaconitic acid were detected at 340 nm. For ITA detection, 2 × 10^4^ BV2 cells were used. The concentration was determined at 450 nm after filtration through glass fiber and addition of the required working solution.

### Total RNA extraction, Transcript Sequencing, and cDNA Library Construction

To further explore how VISTA regulates microglia function, cells were divided into three groups: a control group, an OGD/R model group, and an overexpression VSIR + OGD/R group. Total RNA was extracted and subjected to transcriptomic sequencing. RNA isolation and purification were performed using TRIzol reagent (Invitrogen, Carlsbad, CA, USA) in accordance with the manufacturer’s instructions. The quantity and purity of RNA from each sample were measured using a NanoDrop 2000. RNA integrity was evaluated using an Agilent 2100, with a required RIN number > 7.0. Poly(A) RNA, extracted from 5 µg of total RNA, underwent two rounds of purification using poly-T oligo-attached magnetic beads. Subsequently, the poly (A) RNA was fragmented into small pieces using divalent cations at elevated temperatures and reverse-transcribed to synthesize cDNA. U-labeled second-stranded DNAs were then generated using *E. coli* DNA polymerase I, RNase H, and dUTP. An A-base was added to the blunt ends of each strand, preparing them for ligation to the indexed adapters. Each adapter, featuring a T-base overhang, was ligated to the A-tailed fragmented DNA. This was followed by size selection using AMPure XP beads. After treatment with heat-labile UDG enzyme, the U-labeled second-stranded DNAs were PCR amplified. The average insert size for the final cDNA library was 300 bp (± 50 bp). Finally, 150 bp paired-end sequencing was performed on an Illumina HiSeq 4000 (LC Bio, China), following the vendor’s recommended protocol [34].

### Differential Expression Analysis and Enrichment Analysis

Datasets were sourced from the GEO database (https://www.ncbi.nlm.nih.gov/geo/) and transcript sequencing. The limma package was utilized to obtain differentially expressed genes (DEGs) [35]. Enrichment analysis of common differential genes was conducted using the cluster Profiler package, including Gene Ontology (GO) and Kyoto Encyclopedia of Genes and Genomes (KEGG) analyses. Gene set enrichment analysis (GSEA) was performed using GSEA software (version 4.1.0). The thresholds used above were *p*-value < 0.05.

### Western Blotting

Total protein was extracted from the right hemisphere or BV2 cells and quantified according to standard procedures. Protein concentration was determined using a BCA protein concentration determination kit (Beyotime Biotechnology, China). Proteins were separated by 12.5% sodium dodecyl sulfate–polyacrylamide gel electrophoresis (PG113, Epizyme Biotech, China) and transferred to a 0.45-µm nitrocellulose membrane (BS-NC-45, Biosharp, China). The membrane was blocked with 5% skim milk and incubated with the primary antibody overnight at 4 °C. After washing three times with TBST, the membrane was incubated with a secondary antibody for 1 h. Following three additional TBST washes, the membrane was treated with enhanced chemiluminescence (ECL) reagent (Biosharp, BL520 A, China) for detection. After visualization, the membrane blots were stripped to remove old primary and secondary antibody using Stripping buffer (P0025B, Beyotime, China) [36]. Then, the membrane can be reprobed with other primary antibodies. Band intensities were quantitatively analyzed using ImageJ software (1.8.0). The primary antibodies were as follows: rabbit anti-VISTA (1:1000, ab300042, Abcam), rabbit anti-ACOD1 (1:1000, 28,436–1-AP, Protintech, China), rabbit anti-phosphor P65 (1:1000, ab76302, Abcam), rabbit anti-p65 (1:1000, ab32536, Abcam), rabbit anti-phosphor IκBα (1:1000, ab133462, Abcam), rabbit anti-IκBα (1:1000, ab76429, Abcam), rabbit anti-GAPDH (1:1000, 10,494–1-AP, Protintech, China), rabbit anti-β actin (1:1000, AC026, Abclonal, China), mouse anti-α Tubulin (1:1000, 66,031–1-Ig, Protintech, China). The second antibodies were as follows: horseradish peroxidase (HRP)–conjugated anti-mouse (1:10,000, SA00001-1, Protintech, China) and anti-rabbit (1:10,000, SA00001-2, Protintech, China).

### Quantitative Real-Time PCR Analysis

Total RNA was extracted from the right hemisphere tissue or BV2 cells using TRIzol reagent (15,596,026, Invitrogen, USA), following the standard protocol. The isolated RNA was then converted to cDNA using reverse transcription kits (MR05101S, Monad, China). The mRNA expression levels were quantified using the PCR system (Bio-Rad, Singapore) with the SYBR green kit (4,913,914,001, Roche). Relative gene expression was normalized to GAPDH. The primer sequences are presented in Table 2.

**Table 2:** The primer sequences in this study

IL-6	forward 5′-TAGTCCTTCCTACCCCAATTTCC-3′
	reverse 5′-TTGGTCCTTAGCCACTCCTTC-3′
IL-10	forward 5′-AGCCTTATCGGAAATGATCCAGT-3′
	reverse 5′-GGCCTTGTAGACACCTTGGT-3′
Tnfα	forward 5′-GGTGCCTATGTCTCAGCCTCTT-3′
	reverse 5′-GCCATAGAACTGATGAGAGGGAG-3′
IL-1β	forward 5′-GAAATGCCACCTTTTGACAGTG-3′
	reverse 5′-GAAATGCCACCTTTTGACAGTG-3′
GAPDH	forward 5′-TGACCTCAACTACATGGTCTACA-3′
	reverse 5′-CTTCCCATTCTCGGCCTTG-3′
VSIR	forward 5′-GGAACCCTGCTCCTTGCTATT-3′
	reverse 5′-TTGTAGATGGTCACATCGTGC-3′
ACOD1	forward 5′-TGCTGCTGCGTCCAAGTTT-3′
	reverse 5′-GGGGCTTAGTCTGAGTGGC-3′

### CCK8 Assay

Cell proliferation was assessed using a Cell Counting Kit-8 (CCK8, Solarbio, China) according to the manufacturer’s instructions. BV2 cells were seeded and cultured in a 96-well microplate, then treated with various stimuli. CCK-8 reagent was added to each well and incubated for 2 h. All experiments were repeated, and absorbance was measured at 450 nm using an enzyme label analyzer, with wells lacking cells serving as blanks. Cell proliferation was expressed as absorbance.

### EDU Staining Assay

The cell proliferation status was assessed using Edu detection kit (RiboBio, China). Following treatment, BV2 cells were incubated with Edu for 2 h and the fixed with 4% paraformaldehyde at room temperature for 20 min. Images were captured using a Nikon fluorescence microscope. EDU-positive cells from different treatment groups were analyzed using ImageJ.

### Statistical Analysis

Statistical significance was determined using Prism (8.2.1). Statistical analysis was performed using Student’s two-tailed *t*-test for comparison between two groups, and one-way analysis of variance (ANOVA) followed by Turkey test for three or more groups. Data were expressed as mean ± SEM, and *p* < 0.05 was considered statistically significant. All experiments were performed in triplicates.

## Results

### Identification of Differentially Expressed Immune Checkpoint Genes

**Fig. 1:**
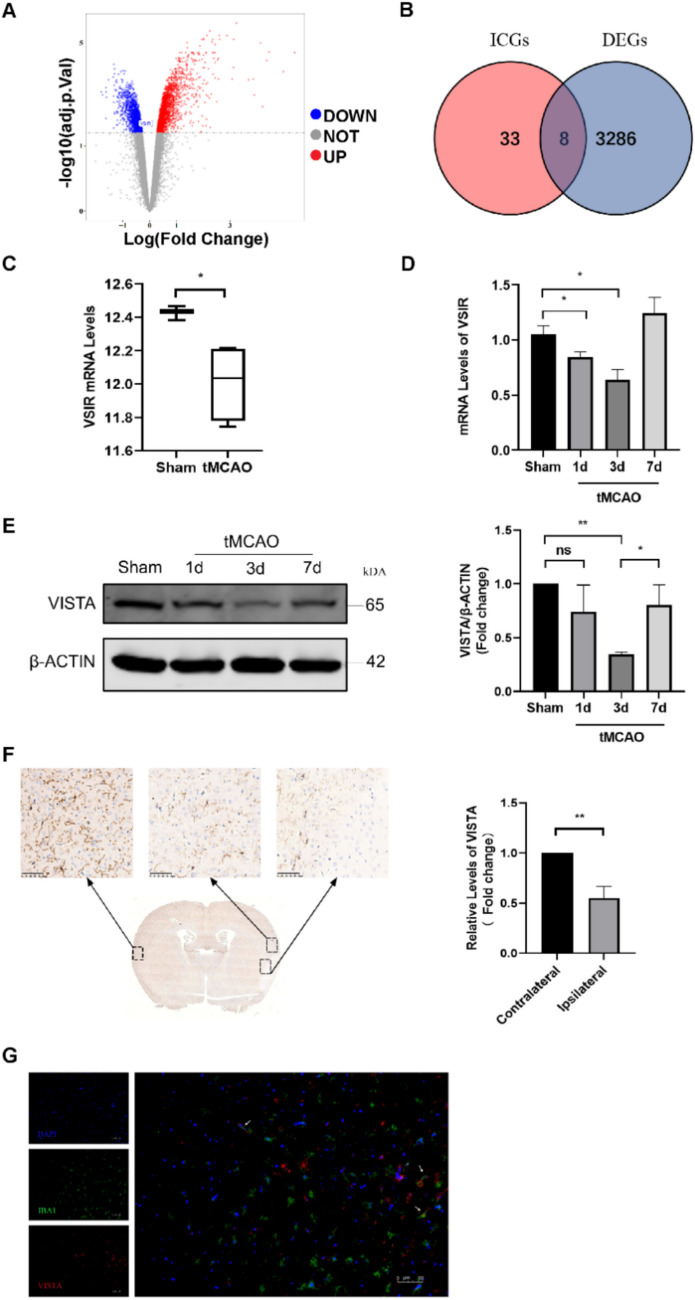
Expression of VISTA in tMCAO mice. **A** Volcano plot to identify DEGs in GSE77986. Red dots represent the upregulated genes and blue dots denote the downregulated genes, with thresholds of |log_2_FC|> 0 and adjusted *p* < 0.05. **B** Venn diagram displaying the intersection of DEGs and ICGs. **C** The expressions and of VSIR in GSE77986.** D** qRT-PCR analyses of VSIR in tMCAO mice. **E** Representative western blotting bands and densitometric quantifications of VISTA in tMCAO mice. **F** Representative immunohistochemistry and densitometric quantifications of VISTA in tMCAO. **G** Representative images of co-expression of Iba1 (green) and VISTA (red) in mouse brain. *p < 0.05; **p < 0.01

GSE77986 contained genes expression data from microglia isolated from five tMCAO model mice and three mice with sham surgery. A total of 3294 DEGs were identified, comprising 1470 downregulated and 1824 upregulated (Fig. 1A). The “homologene” R package was used to convert 47 immune checkpoint genes (ICGs) into 41 mouse homologous genes. The intersection of these DEGs and mouse homologous ICGs was used to identify DE-ICGs. Eight such genes, including VSIR, CD244, CD274, CD80, ICOS, LAG3, PDCD1, and TNFRSF14, were found (Fig. 1B). Compared to the sham group, CD244, CD274, and PDCD1 were upregulated in the tMCAO group, whereas CD80, ICOS, LAG3, TNFRSF14, and VSIR were decreased (Fig.S3). Of these, VSIR, the gene with the highest level, was selected for further analysis. VSIR expression was significantly reduced in the tMCAO group compared with the sham group (Fig. 1C).

### VISTA Decreases in the Peri-infarct Cerebral Tissue After tMCAO

In line with database findings, Western blotting (WB) results indicated a decrease in VISTA, reaching its lowest point at 3 days post-reperfusion, followed by a gradual increase at 7 days (Fig. 1E). A reduction in VSIR was also detected in the peri-infarct cortex by qRT-PCR, showing a slight rise to levels higher than those in sham mice at 7 days (Fig. 1D). Interestingly, immunofluorescence results showed that VISTA is primarily expressed by microglia (Fig. 1G). Its levels around the infarction were declining, with a more significant decrease in the infarction area compared to the contralateral side (Fig. 1F). These results suggest that the dynamic changes in VISTA following tMCAO could contribute to CIRI and that upregulation of VISTA might offer therapeutic benefits.

### VISTA Alleviates Neurological Deficits and Reduces Acute Injury in CIRI.

**Fig. 2:**
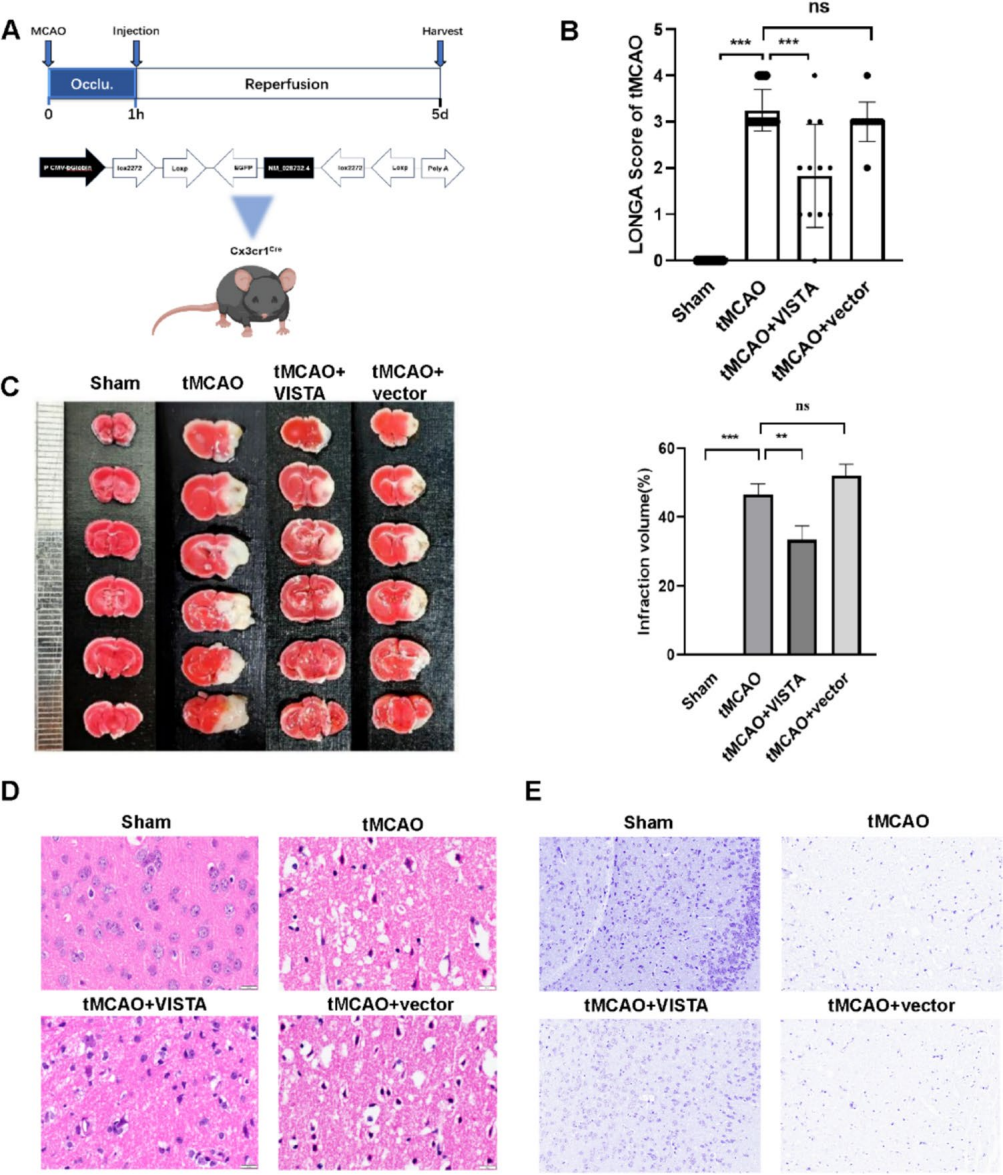
VISTA ameliorated CIRI. **A** Experimental protocol of the present research.** B** Longa scores of tMCAO (*n* = 12). **C** Representative TTC staining and infarction volume of tMCAO. **D** Representative hematoxylin and eosin (H&E) stain of tMCAO. **E** Nissl staining of tMCAO. **p < 0.01; ***p < 0.001

Experimental design is shown as Fig. 2A. Compared to the tMCAO group, VISTA significantly promoted neurological function, as evidenced by the LONGA score (Fig. 2B). TTC staining revealed a substantial reduction in infarct volume due to VISTA (Fig. 2C). VISTA treatment markedly alleviated neuronal and glial cell necrosis, nuclear condensation, and destruction of cell membranes and structures (Fig. 2D, E). These findings demonstrate VISTA’s effectiveness in mitigating CIRI.

### VISTA Inhibits Microglia Inflammation in tMCAO

**Fig. 3:**
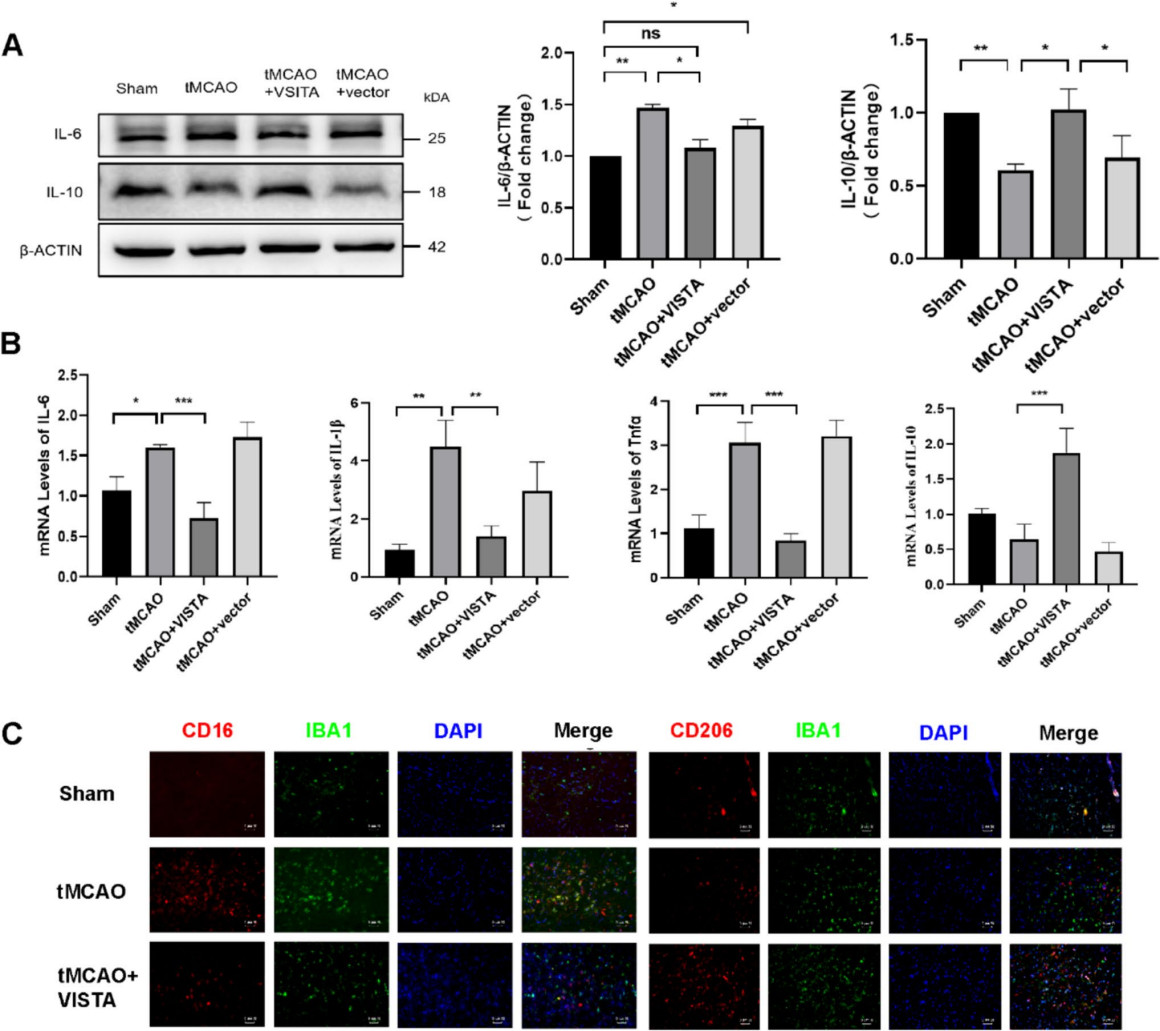
Effect of VISTA on microglia inflammation post-tMCAO. **A** Representative WB bands and densitometric quantifications of IL-6 and IL-10. β-ACTIN was used as a control. **B** qRT-PCR analyses of IL-6, TNFα, IL-1β, and IL-10 of tMCAO. **C** Representative immunofluorescence images of mouse brain tissue. DAPI (blue)/IBA1 (green)/CD16 or CD206 (red). *p < 0.05; **p < 0.01; ***p < 0.001

VISTA decreased IL-6, while increasing IL-10 expression at 5 days post-tMCAO, thereby exerting anti-inflammatory effects (Fig. 3A). These findings were corroborated by qRT-PCR, which showed decreased mRNA levels of IL-6, IL-1β, and TNFα, and increased levels of IL-10 after tMCAO (Fig. 3B). To identify and characterize microglial subtypes, immunofluorescence assays were performed. The number of Iba1 + CD16 + cells significantly increased, whereas the number of Iba1 + CD206 + cells decreased after tMCAO, indicating the activation of the M1 subtype. After VISTA treatment, the number of Iba1 + CD16 + cells decreased, while the number of Iba1 + CD206 + cells increased, suggesting the polarization toward the M2 subtype (Fig. 3C). In conclusion, VISTA alleviated CIRI by inhibiting microglial inflammation.

### VISTA Restrains BV2 Inflammatory Responses and Inhibits the Proliferation After OGD/R

**Fig. 4:**
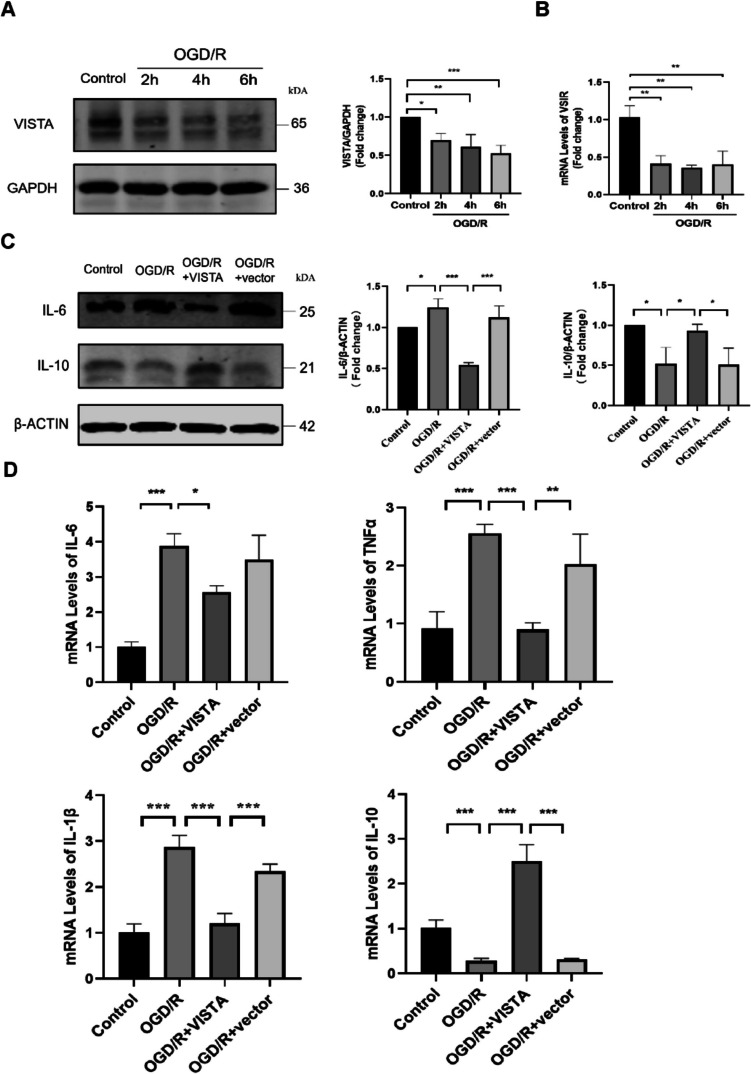
Expression of VISTA in BV2 and its effect post-OGD/R. **A** Representative WB bands and densitometric quantifications of VISTA in BV2 for different OGD. GAPDH was used as a control. **B** QRT-PCR analyses of VSIR in BV2 for different OGD. **C** Representative WB bands and densitometric quantifications of IL-6 and IL-10. β-Actin was used as a control. **D** QRT-PCR analyses of IL-6, IL-1β, TNFα, and IL-10. *p < 0.05; **p < 0.01; ***p < 0.001; ****p < 0.0001

To validate VISTA’s effect, we employed an in vitro OGD/R model, focusing on OGD 2 h, 4 h, and 6 h followed by reperfusion 24 h. VISTA levels were found to progressively decrease, reaching the most significant point at OGD/R6 h (Fig. 4A). qRT-PCR analysis confirmed this trend, indicating a significant reduction in VSIR levels from as early as 2 h post-OGD/R (Fig. 4B). The expression levels of VISTA were increased in BV2 which was transfected with plasmids, proving that the transfection technique was successful (Fig.S4). Remarkably, overexpressed VISTA significantly reduced IL-6 production and elevated IL-10 levels compared to the OGD/R group (Fig. 4C). QRT-PCR results showed inhibition of IL-6, TNFα, IL-1β, and upregulation of IL-10 (Fig. 4D). These results demonstrated that VISTA suppressed pro-inflammatory capabilities of BV2 cells post-OGD/R in vitro, enhancing their anti-inflammatory potential, consistent with in vivo results.

**Fig. 5:**
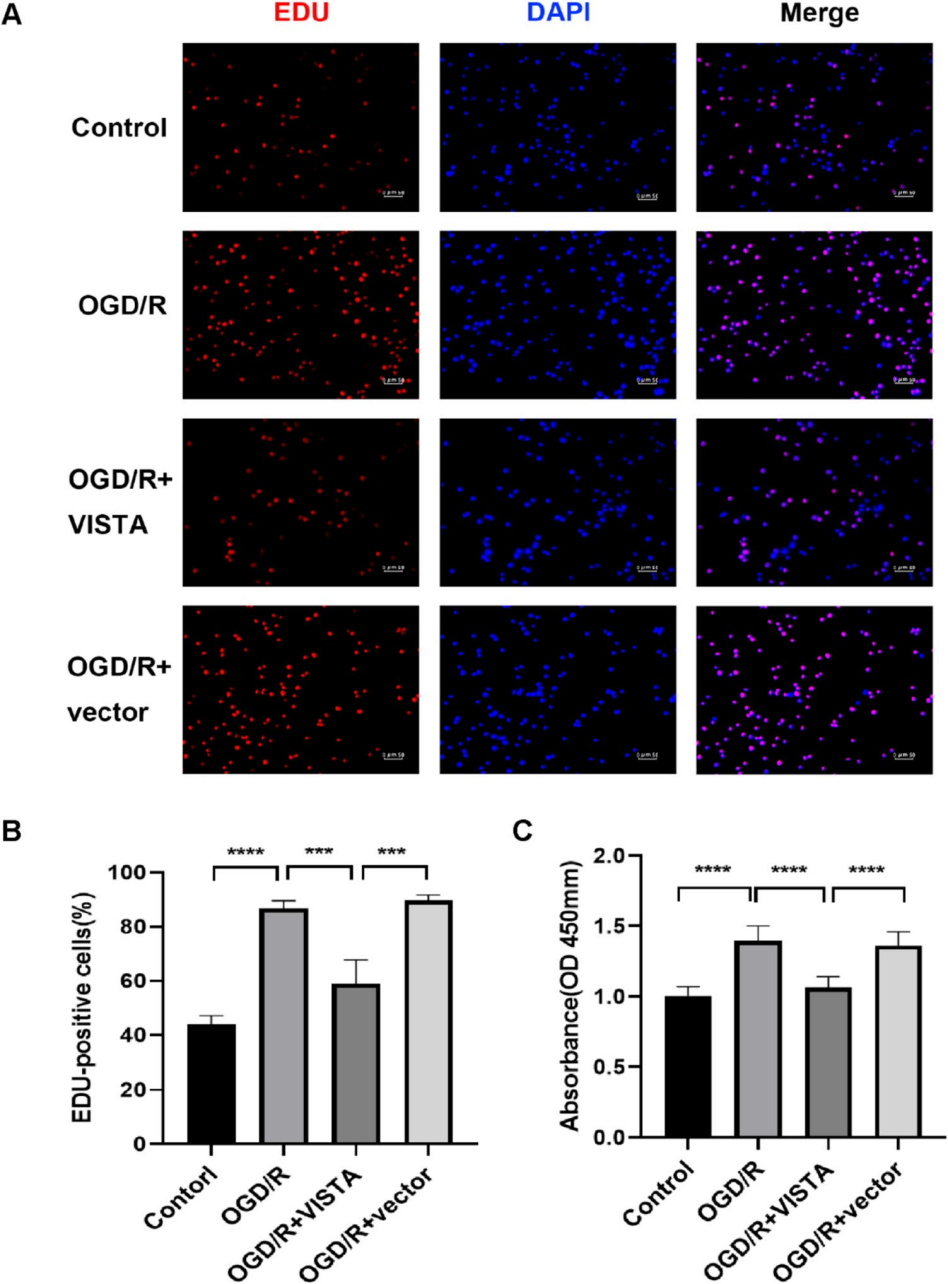
**A**, **B** Representative Edu staining and positive cell proportion of BV2 post-OGD/R. **C** Representative CCK8 of BV2 post-OGD/R. ***p < 0.001; ****p < 0.0001

We performed CCK-8 and Edu assay to test BV2 proliferation. VISTA significantly curtailed the proliferation rate of BV-2 cells post-transfection compared to the OGD/R group (Fig. 5C). Edu staining further confirmed VISTA’s inhibitory effect on cell proliferation (Fig. 5A, B).

### VISTA Regulates the TCA Cycle Metabolites of BV2

**Fig. 6:**
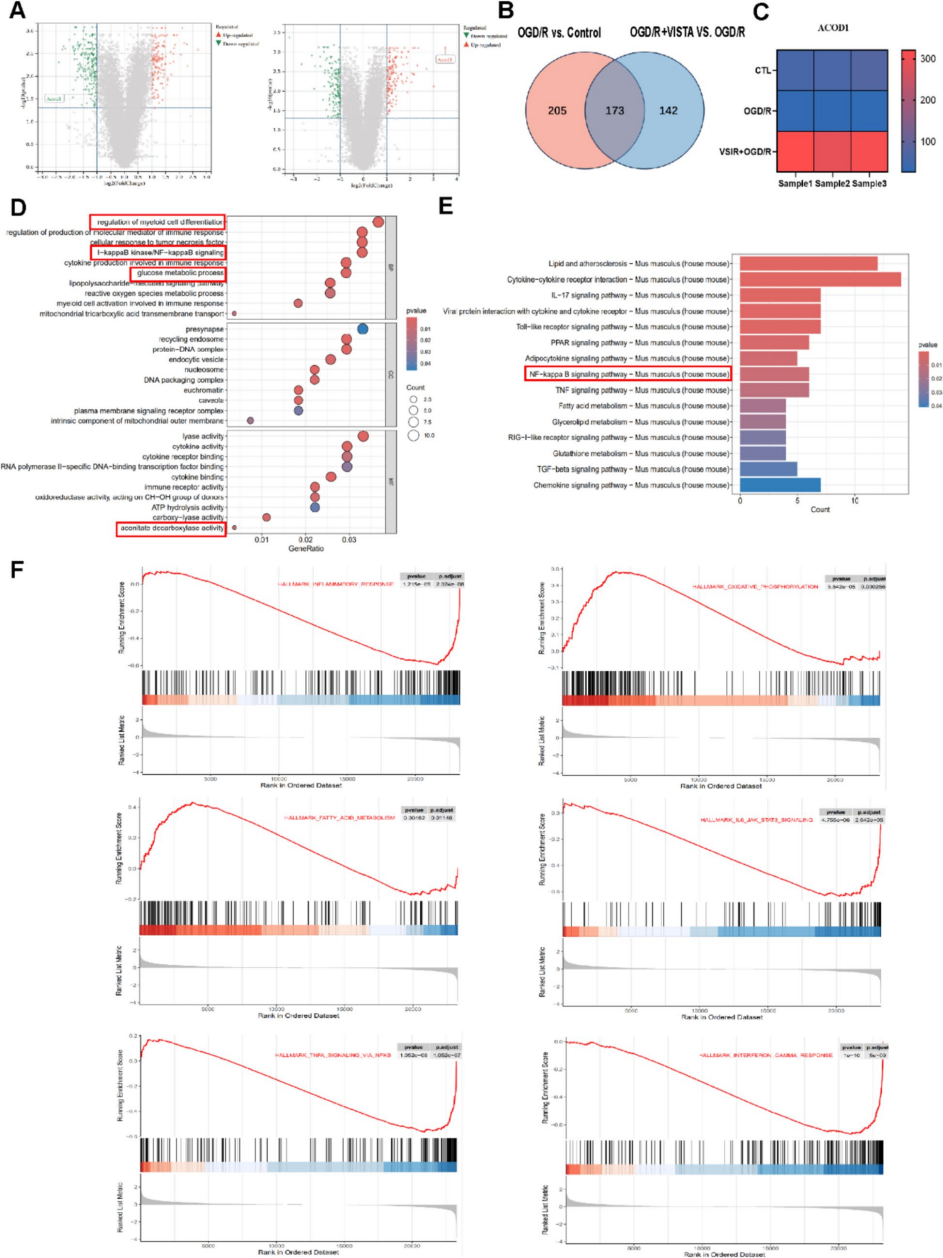
Identification of VISTA downstream pathway by RNA-seq analysis. **A** Volcano plot to identify differentially expressed genes in “OGD/R vs. control” and “OGD/R + VISTA vs. OGD/R.” Red dots represent the upregulated genes and green dots denote the downregulated genes, with thresholds of |log2 FC|> 2 and adjusted *p* < 0.05. **B** Venn diagram displaying the intersection of DEGs from the two groups above.** C** Heatmap displaying the expressions of ACOD1 in control, OGD/R, and VSIR + OGD/R groups. Red bricks indicate the higher expression and blue bricks indicate the lower expression. **D** GO enrichment analysis enriched by DEGs from OGD/R + VISTA vs. OGD/R. **E** KEGG pathway enrichment analysis enriched by DEGs from OGD/R + VISTA vs. OGD/R. **F** GSEA enrichment analysis enriched by DEGs from OGD/R + VISTA vs. OGD/R.* p* < 0.05

To elucidate VISTA’s mechanism, transcriptome sequencing and analysis were employed. DEG analysis was conducted comparing the OGD/R versus the control group, and OGD/R + VISTA versus OGD/R respectively. A total of 378 DEGs were identified between the OGD/R and control groups (155 upregulated and 223 downregulated), and 315 DEGs were noted between the OGD/R + VISTA and OGD/R groups (153 upregulated and 162 downregulated) (Fig. 6A). Intersection of DEGs between these groups revealed 173 significantly different genes (Fig. 6B). Among these, ACOD1’s differential expression was the most notable. ACOD1 was downregulated in the OGD/R group, while upregulated in the VIST + OGD/R group (Fig. 6C). To determine the biological functions and pathways for VISTA, DEGs between OGD/R + VISTA and OGD/R groups were subjected to Gene Ontology (GO) enrichment and Kyoto Encyclopedia of Genes and Genomes (KEGG) pathway analyses. As expected, DEGs were significantly involved in various immune-related biological processes such as regulation of molecular mediator production in immune response, cytokine production, and myeloid cell differentiation and activation. Interestingly, DEGs were also obviously enriched in metabolism-related biological process, including glucose metabolism (Fig. 6D). KEGG pathway analyses indicated involvement in cytokine-cytokine receptor interaction, IL-17 signaling pathway, and viral protein interaction with the NFκB signaling pathway (Fig. 6E). Ultimately, Gene Set Enrichment Analysis (GSEA) demonstrated that VISTA promoted oxidative phosphorylation and fatty acid metabolism in BV2 cells, inhibited the BV2 NF-κB pathway, reduced IL6 production, and suppressed inflammation (Fig. 6F). These results suggested VISTA inhibits the NF-κB pathway and alleviates the inflammatory in BV2 cells through ACOD1-mediated immunometabolism remodeling.

### VISTA Regulates BV2 TCA by ACOD1

**Fig. 7:**
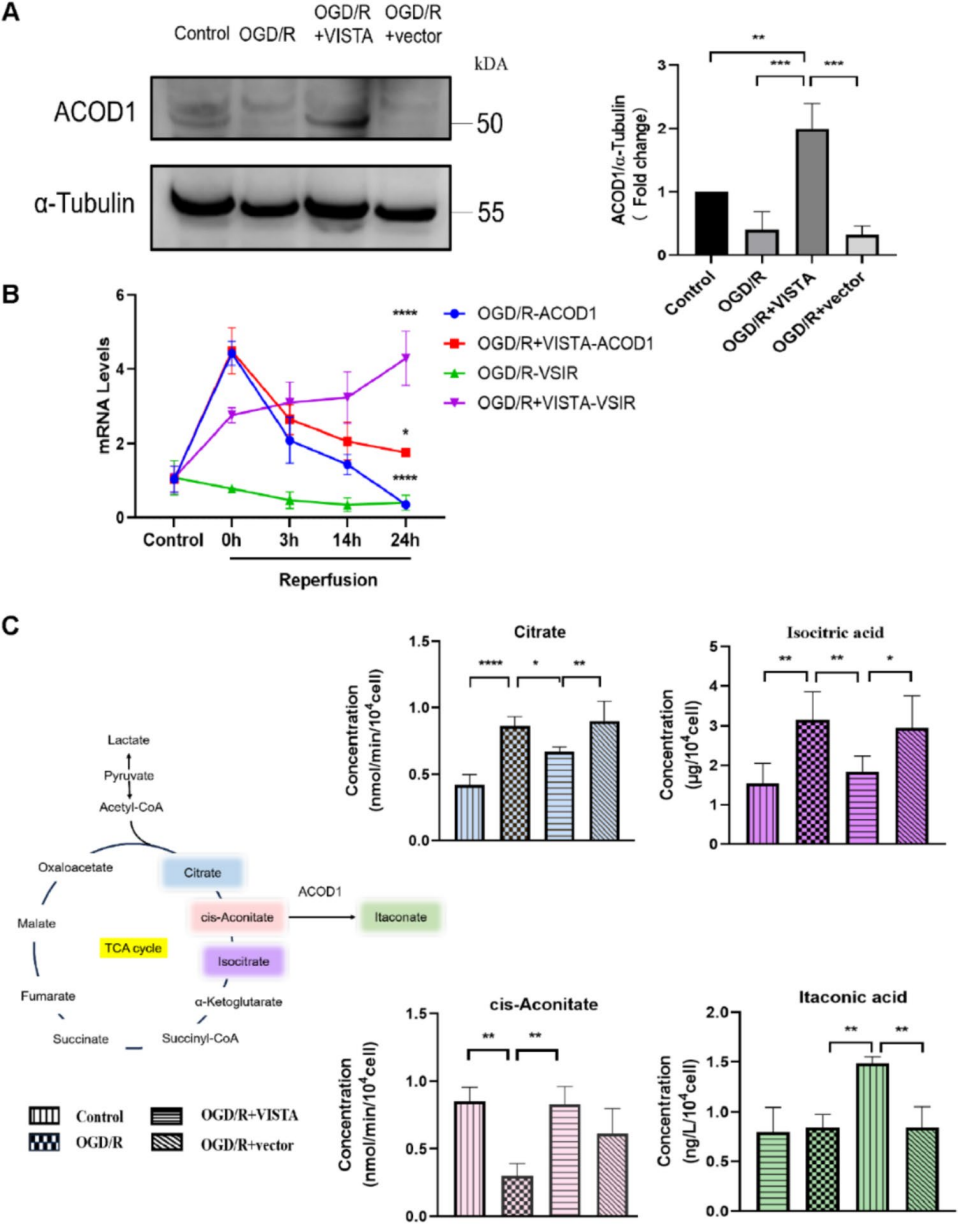
Effect of VISTA on ACOD1 and TCA cycle of BV2 in vitro. **A** Representative WB bands and densitometric quantifications of ACOD1 in BV2. Tubulin α used as a control. **B** qRT-PCR analyses of ACOD1 in BV2. **C** The concentration of citrate, iso-citric acid, cis-aconitate, and ITA detecting by enzyme label analyze. *p < 0.05; **p < 0.01; ***p < 0.001; ****p < 0.0001

As shown in the Fig. 7B, ACOD1 mRNA levels notably increased post-OGD, but gradually declined with the extension of reperfusion time. At 24 h post-reperfusion, ACOD1 levels dropped below those in the control group. The highest VSIR levels were observed at 24 h post-reperfusion after transfection and concurrently ACOD1 levels were higher, suggesting that VSIR augmented ACOD1 expression. Further, WB results confirmed that VISTA upregulated ACOD1 compared with the OGD/R group (Fig. 7A). VISTA was found to modulate key enzymes in the TCA cycle. Specifically, VISTA reduced the levels of citrate and isocitric acid while elevating cis-aconitate and ITA production (Fig. 7C). This indicates that VISTA influences the BV2 TCA cycle through ACOD1 mediation.

### VISTA Inhibits BV2 Inflammation by Regulating the ACOD1-Mediated IκBα/NF-κB Pathway

**Fig. 8:**
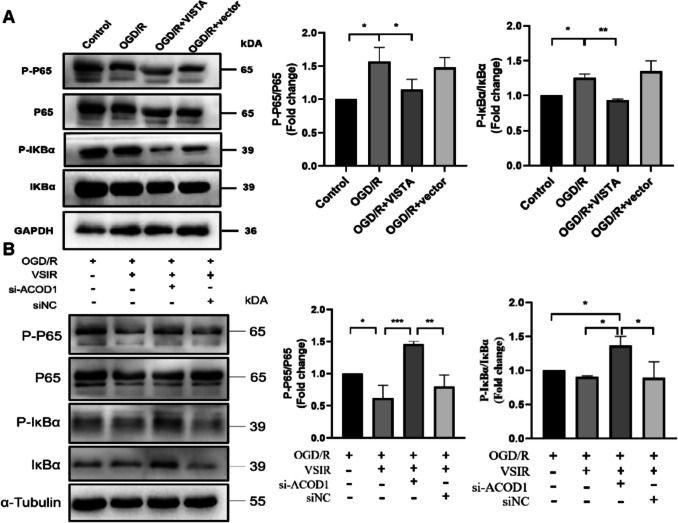
Effect of ACOD1-mediated IκBA/NF-κB pathway on VISTA in vitro. **A** Representative WB bands and densitometric quantifications of IκBA/NF-κB in BV2. GAPDH was used as a control. **B** Representative WB bands and densitometric quantifications of I IκBA/NF-κB in BV2. Α-Tublin was used as a control.*p < 0.05; **p < 0.01; ***p < 0.001

We investigated whether the ACOD1-mediated IκBα/NF-κB pathway is a downstream signaling cascade of VISTA. Consistent with previous findings, activation of the IκBα/NF-κB pathway was observed post-OGD/R. VISTA significantly decreased the phosphorylation of P65 and IκBα compared to the OGD/R group (Fig. 8A), indicating VISTA’s inhibitory effect on the IκBα/NF-κB pathway. To further explore the role of the ACOD1-dependent IκBα/NF-κB pathway in VISTA’s protective mechanism, si-ACOD1 was transfected into BV2 cells. Notably lower ACOD1 levels in the si-ACOD1 group compared to the control group confirmed successful transfection (Fig.S5). Additionally, compared to VISTA overexpression alone, ACOD1 knockdown markedly diminished VISTA’s inhibitory effects on the IκBα/NF-κB pathway (Fig. 8B).

**Fig. 9:**
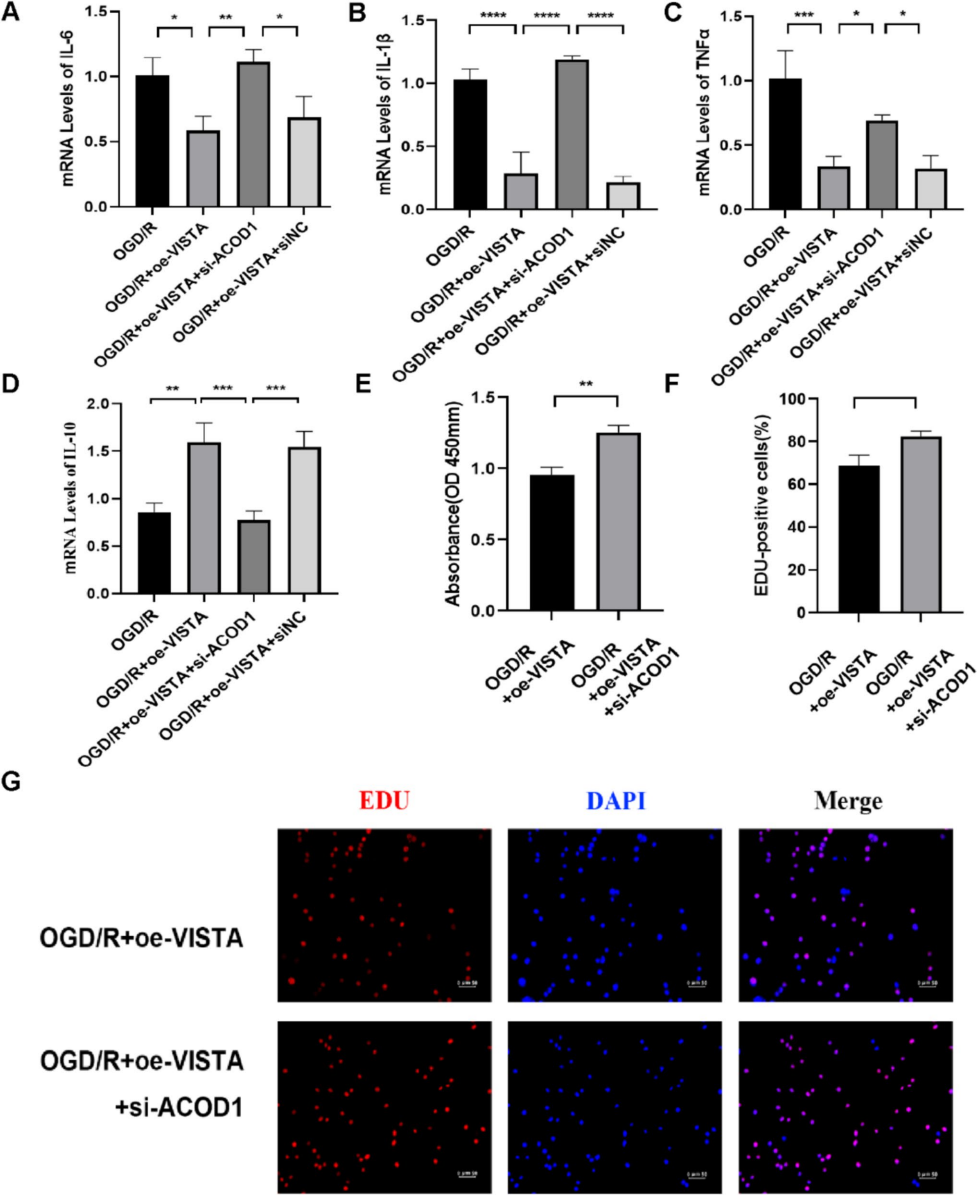
Effect of ACOD1-mediated IκBA/NF-κB pathway on BV2 in vitro. **A**–**D** QRT-PCR analyses of inflammatory factor in BV2. **E** Representative CCK8 of BV2. **F**,** G** Representative Edu stain and positive cells proportion of BV2. *p < 0.05; **p < 0.01; ***p < 0.001. ****p < 0.0001

Interestingly, following ACOD1 knockdown, VISTA was unable to reduce IL-6, TNFα, and IL-1β levels or enhance IL10 production (Fig. 9A–D). CCK8 assays confirmed that VISTA’s suppressive effect on BV2 proliferation was nullified following ACOD1 knockdown (Fig. 9E). BV2 proliferation was more pronounced post-ACOD1 knockdown compared to VISTA overexpression alone, as shown by the Edu assay (Fig. 9F, G). In summary, VISTA mitigates the inflammatory response and proliferation of BV2 cells by modulating the ACOD1-mediated IκBα/NF-κB pathway.

## Discussion

Increasing evidence suggests that cerebral ischemia–reperfusion initiates a series of pathological cascades, including excitotoxicity, oxidative stress, inflammation, apoptosis, and cell death [37]. Microglia, the resident immune cells of the brain, become activated after reperfusion, with their mitochondrial functions undergoing dynamic changes in response to stimuli [38]. ACOD1 catalyzes the formation of itaconic acid, a TCA cycle intermediate, and reduces the inflammatory response of immune cells. In our study, we observed a decrease in VISTA expression following CIRI. Overexpressing VISTA in microglia of Cx3cr1^Cre^ mice using the tMCAO model reduced lesion volume and neurological function deficit. Notably, VISTA induced ACOD1 expression, altering the TCA cycle metabolites in microglia by promoting ITA production after OGD/R stimulation. VISTA alleviated inflammatory cytokine production and inhibited cell proliferation by ACOD1/IκBα/NF-κB.

Immune checkpoint molecules, acting as gatekeepers of immune responses, significantly impact cellular immunity by limiting inflammatory response and maintaining physiologic tissue conditions [39]. Disruption of immune checkpoint can lead to autoimmunity or hinder specific immune responses [40]. Currently, 47 classical immune checkpoints have been identified in humans. Antibodies targeting CTLA-4 and PD-1, B7 family co-inhibitory receptors, have elicited durable clinical outcomes in previously refractory cancer types and are considered a breakthrough in cancer therapy [41]. In our study, 8 immune checkpoints associated with acute ischemic stroke (AIS) were identified: VSIR, CD244, CD274, CD80, ICOS, LAG3, PDCD1, and TNFRSF14. Previous studies indicate that CD274, also known as PD-L1, exacerbates inflammation in stroke, and treatments with anti-PD-L1 antibodies control CNS inflammation [42, 43]. Conversely, PD-1 signaling, a major pathway protecting against CNS inflammation in MCAO, likely plays a crucial role in reducing stroke-associated TLR2- and TLR4-mediated neurotoxic factor release by activated CNS microglia [44]. ICOS knockout protects brain tissues from ischemia injuries and inhibits T cell–induced cytokines [45]. Similarly, LAG3, a microglial checkpoint, has been linked to suicide in bipolar disorder patients due to reduced LAG3 expression [46]. Specific single-nucleotide polymorphisms (SNPs) of the LAG3 gene have been also related to Parkinson’s disease occurrence especially in the female population [47]. The V domain–containing immunoglobulin suppressor of T cell activation (VISTA), also termed PD-1 homolog (PD-1H), C10orf54, belongs to the B7 family [48]. In the CNS, VISTA is primarily expressed by microglia, with expression levels akin to signature genes. VISTA decreases in response to a range of TLR agonist and the stimulation with various pro-inflammatory compounds in vitro[18, 49]. VISTA expression is dynamically regulated under inflammatory conditions, as evidenced by its reduction at all stages of EAE [20]. However, the relationship between VISTA and microglia in ACI has not been studied. The present study confirmed that the levels of VISTA are significantly decreased both in peri-infarct cerebral tissue post-tMCAO and in BV2 with OGD/R. This finding aligns with previous studies and our data analysis.

Previous evidences showed that microglia are double-edged sword in nerve repair, as resting microglia can adopt either classically activated (M1) or alternatively activated (M2) phenotypes [50, 51]. M1-type microglia produce pro-inflammatory cytokines such as IL-1β, IL-6, and TNF-α, iNOS, as well as MHC-II; chemokines such as CCL2 and CXCL9; and large amounts of ROS. In contrast, M2-type microglia, which can be further categorized into M2a, M2b, and M2c subclasses, exhibit neuroprotective characteristics. M2a microglia release anti-inflammatory factors such as IL-10 and IGF-1; upregulate Arg-1, CD206, and Ym1 expression; inhibit NF-κB; and enhance phagocytosis [52, 53]. Recent research has identified eight distinct microglial subpopulations post I/R injury, categorized into four major groups: Mic_home (homeostatic microglia), Mic_pre (preliminary active microglia), Mic_M1L (M1-like-polarization microglia), and Mic_np (neuropeptide-secretion microglia). Mic_home and Mic_pre exist before ischemic stroke, while Mic_M1L types 1 and 2, which show significant activation of inflammatory genes, are primarily found in post-stroke samples [54, 55]. As research advances, microglial classification is expected to become more refined and scientific. This study primarily focuses on microglia’s role in regulating inflammation and their proliferative ability. We observed a decrease in the production of pro-inflammatory cytokines IL-6, TNFα, and IL-1β and an increase in anti-inflammatory factors IL-10 following VISTA overexpression after CIRI. Our results demonstrate that VISTA modulates microglial function in CIRI by inhibiting pro-inflammation, reducing proliferation, and enhancing anti-inflammation both in vivo and in vitro. VISTA mitigates behavioral deficits, diminishes infarct size, and improves pathological damage in brain tissue post-reperfusion. These findings suggest VISTA as a potential therapeutic approach for CIRI.

**Fig. 10:**
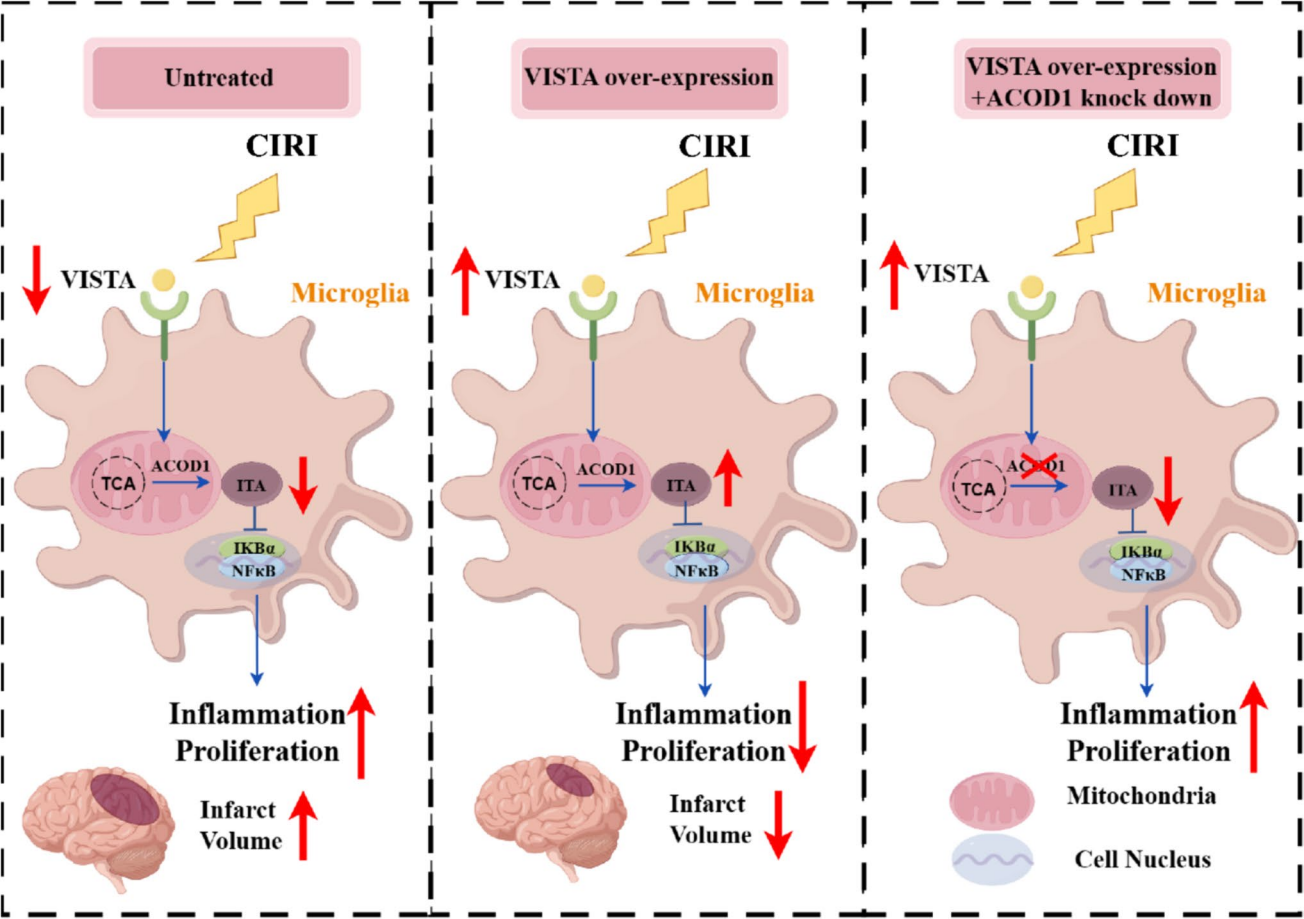
The protective role of VISTA in microglia during tMCAO

Metabolism constitutes a network of biochemical reactions that convert nutrients into metabolites, enabling cells to generate the energy, redox equivalents, and macromolecules (including proteins, lipids, DNA, and RNA) required for survival and maintenance of cellular functions [56]. The TCA cycle, central to all mammalian cell types, has been linked to microglial function [57]. A shift to the M1 phenotype in cells is often associated with a transition from oxidative phosphorylation to aerobic glycolysis for energy production [58, 59]. Metabolic changes are known to modulate the homeostatic functions of microglia [60, 61]. Our study suggests that VISTA influences microglial metabolism, including fatty acid and glucose metabolism, as revealed by RNA sequencing. ACOD1 and its metabolite ITA have garnered attention for their anti-inflammatory roles in macrophages and microglia [62, 63]. The ACOD1/ITA axis in myeloid cells is induced by stimuli such as LPS and infection. Previous research suggested that ischemic stroke or LPS stimuli increased ACOD1 [12, 13]. However, our current findings reveal that ACOD1 initially increases significantly at the onset of reperfusion and gradually decreases as reperfusion progresses. After 24 h of reperfusion, ACOD1 levels reach their lowest point, lower than those in the control group, suggesting a dynamic process. Previous studies have shown that a deficiency in ACOD1 exacerbates ischemic injury. In line with this, we found that the neuroprotective and TCA-regulating functions of VISTA are counteracted by ACOD1 knockdown. Therefore, we demonstrate that VISTA’s protective effect against CIRI is mediated by ACOD1. Further research has revealed ITA’s roles in immunomodulation as an Nrf2 activator and a regulator of the ATF3/IκBζ axis [64]. Our study found that ACOD1 affects the phosphorylation of IκBα/NF-κB and that the ACOD1/IκBα/NF-κB is a downstream pathway of VISTA (Fig. 10).

The present study had certain limitations. The ligand of VISTA in CNS has not been well studied, which is needed to be evaluated in future studies. In conclusion, we report for the first time that VISTA protects against CIRI and inhibits microglial inflammation by ACOD1/IκBα/NF-κB. These findings indicate VISTA as a novel adjunct therapeutic strategy for the management of CIRI. Given the advancements in immune checkpoint therapy, the translation of VISTA to clinical practice appears promising for future application.

## Supplementary Information

Below is the link to the electronic supplementary material.

Supplementary file1 (ZIP 637 KB)

## Data Availability

No datasets were generated or analysed during the current study.
