# Supplementary material for: VISTA Alleviates Microglia-Mediated Neuroinflammation After Cerebral Ischemia–Reperfusion Injury via Regulating ACOD1/Itaconic Acid Metabolism
Source: Mol Neurobiol. 2025 Jun 19;62(10):13430–48. doi: 10.1007/s12035-025-05106-x (PMC12433375; doi:10.1007/s12035-025-05106-x)
Supplement: Supplementary file 1 — Supplementary file1 (ZIP 637 KB) [file 12035_2025_5106_MOESM1_ESM.zip › Fig S4.pdf]

Article title	VISTA Alleviates Microglia-mediated Neuroinflammation after Cerebral Ischemia-Reperfusion Injury via Regulating ACOD1/ Itaconic acid metabolism.
Journal name	Molecular Neurobiology
Author names	Yilei Sun, Dan Liu, Yanchen Liu, Lijun Chi*
Affiliation and e-mail address of the corresponding author.	Department of Neurology, The First Affiliated Hospital of Harbin Medical University CLJ3787@163.com

Supplementary Information (SI) 4

The VISTA overexpression plasmids or vector PCDNA3.1 were transfected into BV2.The expression levels of VISTA were increased in BV2 which was transfected with plasmids, proving that the transfection technique was successful.

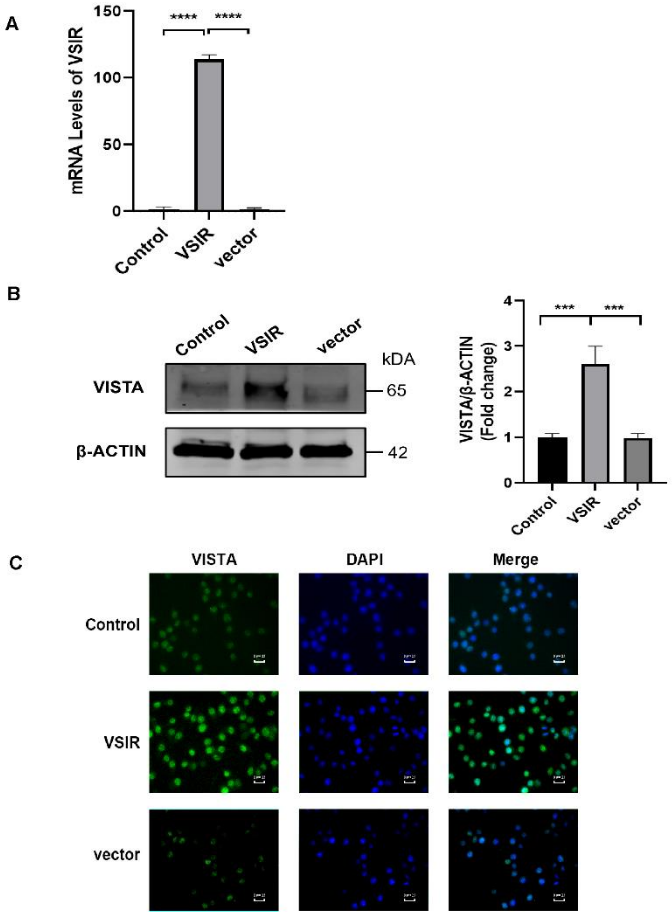

Fig.S4 Transfection efficiency of VISTA overexpression plasmids. **A** qRT-PCR analyses of VSIR in BV2. **B** Representative western blotting bands and densitometric quantifications of VISTA in BV2. ***$p < 0.001$; ****$p < 0.0001$. **C** Representative images of immunofluorescence staining.
