# Supplementary material for: VISTA Alleviates Microglia-Mediated Neuroinflammation After Cerebral Ischemia–Reperfusion Injury via Regulating ACOD1/Itaconic Acid Metabolism
Source: Mol Neurobiol. 2025 Jun 19;62(10):13430–48. doi: 10.1007/s12035-025-05106-x (PMC12433375; doi:10.1007/s12035-025-05106-x)
Supplement: Supplementary file 1 — Supplementary file1 (ZIP 637 KB) [file 12035_2025_5106_MOESM1_ESM.zip › Fig S5.pdf]

Article title	VISTA Alleviates Microglia-mediated Neuroinflammation after Cerebral Ischemia-Reperfusion Injury via Regulating ACOD1/ Itaconic acid metabolism.
Journal name	Molecular Neurobiology
Author names	Yilei Sun, Dan Liu, Yanchen Liu, Lijun Chi*
Affiliation and e-mail address of the corresponding author.	Department of Neurology, The First Affiliated Hospital of Harbin Medical University CLJ3787@163.com

Supplementary Information (SI) 5

The si-ACOD1 or si-NC was transfected into BV2.The expression levels of ACOD1 were decreased in BV2 which was transfected, proving that the transfection technique was successful.

A

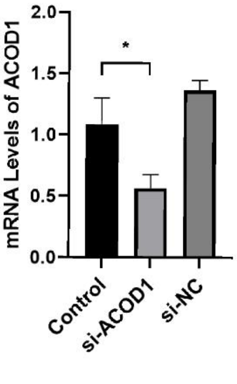

B

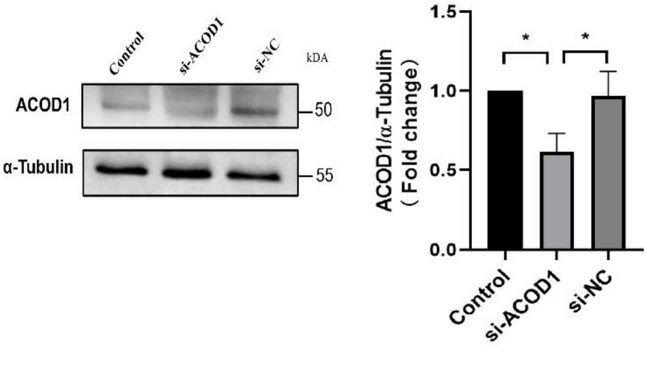

Fig.S5 Knockdown efficiency of si-ACOD1. **A** qRT-PCR analyses of ACOD1 in BV2. **B** Representative western blotting bands and densitometric quantifications of ACOD1 in BV2. *$p < 0.05$.
