# Supplementary material for: VISTA Alleviates Microglia-Mediated Neuroinflammation After Cerebral Ischemia–Reperfusion Injury via Regulating ACOD1/Itaconic Acid Metabolism
Source: Mol Neurobiol. 2025 Jun 19;62(10):13430–48. doi: 10.1007/s12035-025-05106-x (PMC12433375; doi:10.1007/s12035-025-05106-x)
Supplement: Supplementary file 1 — Supplementary file1 (ZIP 637 KB) [file 12035_2025_5106_MOESM1_ESM.zip › Fig S1.pdf]

Article title	VISTA Alleviates Microglia-mediated Neuroinflammation after Cerebral Ischemia-Reperfusion Injury via Regulating ACOD1/ Itaconic acid metabolism
**Journal name**	Molecular Neurobiology
**Author names**	Yilei Sun, Dan Liu, Yanchen Liu, Lijun Chi*
**Affiliation and e-mail address of the corresponding author.**	Department of Neurology, The First Affiliated Hospital of Harbin Medical University CLJ3787@163.com

Supplementary Information (SI) 1

All male Cx3cr1^{Cre} mice involved in the experiments were heterozygous. The mouse breeding method involved positive cross-mating. To confirm the presence of the Cre sequence, genomic DNA extracted from the tail was amplified using the following primers. Finally, heterozygous mice were selected for use in the experiments

Primers for Region1 PCR:	Mutant: 508 bp
P1:	5’-GGATGAGTGAAGACAAAATCTAGTTCC-3’
P2:	5’-CTTGCAGGTACAGGAGGTAGTCC-3’
Primers for Region2 PCR:	Wildtype: 307 bp
P1:	5’- GGATGAGTGAAGACAAAATCTAGTTCC-3’
P3:	5’- CCTCAGCAGAATCGTCATACTCAA-3’

Mutant: 508 bp
Heterozygote: 508 bp and 307 bp
Wild type: 307 bp

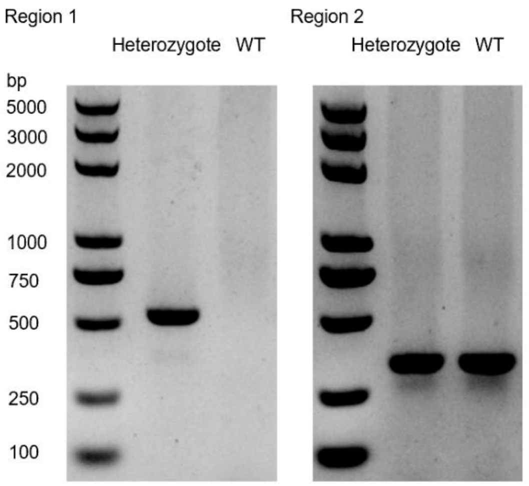

Fig.S1: Genotype identification of Cx3cr1^{Cre} mice.
