# Supplementary material for: VISTA Alleviates Microglia-Mediated Neuroinflammation After Cerebral Ischemia–Reperfusion Injury via Regulating ACOD1/Itaconic Acid Metabolism
Source: Mol Neurobiol. 2025 Jun 19;62(10):13430–48. doi: 10.1007/s12035-025-05106-x (PMC12433375; doi:10.1007/s12035-025-05106-x)
Supplement: Supplementary file 1 — Supplementary file1 (ZIP 637 KB) [file 12035_2025_5106_MOESM1_ESM.zip › Fig S2.pdf]

Article title	VISTA Alleviates Microglia-mediated Neuroinflammation after Cerebral Ischemia-Reperfusion Injury via Regulating ACOD1/ Itaconic acid metabolism.
**Journal name**	Molecular Neurobiology
**Author names**	Yilei Sun, Dan Liu, Yanchen Liu, Lijun Chi*
**Affiliation and e-mail address of the corresponding author.**	Department of Neurology, The First Affiliated Hospital of Harbin Medical University<br>CLJ3787@163.com

Supplementary Information (SI) 2

AAVMG1.2-VSIR or AAVMG1.2-NC was injected intracerebroventricularly into Cx3cr1^{Cre} mice using the method mentioned. Samples were collected five days later. Immunofluorescence staining and Western Blot (WB) analysis results indicated significant increases in VISTA protein levels in microglia post-injection (Fig.S2A,S2C). Elevated levels of VSIR were also observed (Fig. S2B), demonstrating the success of the AAV intervention.

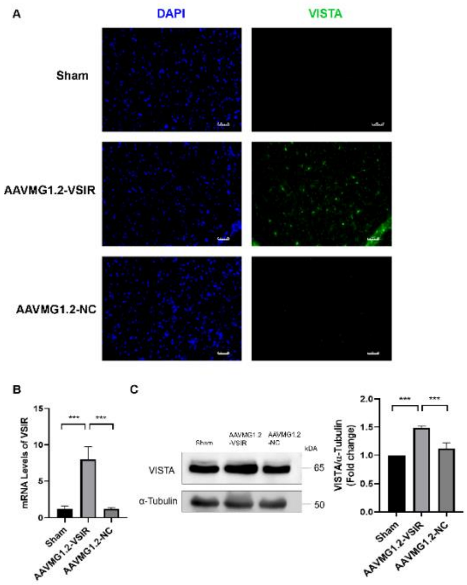

Fig.S2 Transfection efficiency of AAVMG1.2-VSIR. **A** Representative Immunofluorescence of VISTA in Cx3cr1^{Cre} mice post-injection intracerebroventricularly. **B** qRT-PCR analyses of VSIR in Cx3cr1^{Cre} mice post-injection intracerebroventricularly. **C** Representative western blotting bands and densitometric quantifications of VISTA in Cx3cr1^{Cre} mice post-injection intracerebroventricularly. ***$p < 0.001$.
